# Supplementary material for: Interdisciplinary collaboration from diverse science teams can produce significant outcomes
Source: PLoS One. 2022 Nov 29;17(11):e0278043. doi: 10.1371/journal.pone.0278043 (PMC9707800; doi:10.1371/journal.pone.0278043)
Supplement: S1 Appendix — (DOCX) [file pone.0278043.s001.docx]

**S1 Appendix Demographic profiles of the groups from the contributing organisations.**

| **group** | **start** | **members** | **female (%)** | **disciplines** | **countries** |
| --- | --- | --- | --- | --- | --- |
| A-1 | 2014 | 11 | 9 | 5 | 5 |
| A-2 | 2012 | 15 | 33 | 6 | 4 |
| A-3 | 2010 | 12 | 25 | 5 | 2 |
| A-4 | 2014 | 14 | 36 | 3 | 7 |
| A-5 | 2010 | 17 | 18 | 2 | 8 |
| A-6 | 2015 | 20 | 15 | 5 | 10 |
| A-7 | 2011 | 13 | 31 | 6 | 7 |
| A-8 | 2011 | 13 | 8 | 4 | 6 |
| A-9 | 2012 | 17 | 18 | 5 | 7 |
| B-1 | 2011 | 17 | 67 | 9 | 1 |
| B-2 | 2010 | 24 | 0 | 4 | 1 |
| B-3 | 2012 | 18 | 42 | 7 | 2 |
| B-4 | 2010 | 19 | 41 | 10 | 2 |
| B-5 | 2010 | 21 | 24 | 4 | 4 |
| B-6 | 2010 | 15 | 60 | 6 | 2 |
| B-7 | 2010 | 16 | 63 | 8 | 4 |
| C-1 | 2014 | 20 | 40 | 7 | 4 |
| C-2 | 2014 | 22 | 24 | 7 | 1 |
| C-3 | 2012 | 17 | 28 | 9 | 7 |
| C-4 | 2015 | 14 | 41 | 7 | 2 |
| C-5 | 2013 | 20 | 38 | 5 | 3 |
| C-6 | 2015 | 28 | 32 | 8 | 2 |

The organisations and groups have been given codes to anonymise the source.
