# Supplementary material for: Interdisciplinary collaboration from diverse science teams can produce significant outcomes
Source: PLoS One. 2022 Nov 29;17(11):e0278043. doi: 10.1371/journal.pone.0278043 (PMC9707800; doi:10.1371/journal.pone.0278043)
Supplement: S2 Appendix — (DOCX) [file pone.0278043.s002.docx]

**S2 Appendix.** **Profile of publications produced and cited by each group.**

|  | **publications** | | | | | **articles cited** | | |
| --- | --- | --- | --- | --- | --- | --- | --- | --- |
| **Group** | **number** | **disciplinary divisions** | **Shannon diversity** | **average no. authors** | **median citations (August 2020)** | **number** | **disciplinary divisions** | **Shannon diversity** |
| A-1 | 2 | 2 | 0.693 | 10.50 | 12.5 | 79 | 2 | 0.287 |
| A-2 | 5 | 2 | 0.673 | 13.20 | 19 | 204 | 9 | 1.249 |
| A-3 | 9 | 2 | 0.530 | 11.44 | 22 | 591 | 7 | 0.740 |
| A-4 | 3 | 1 | 0 | 19.00 | 4.5 | 99 | 6 | 0.716 |
| A-5 | 24 | 2 | 0.377 | 8.54 | 41 | 1294 | 11 | 0.834 |
| A-6 | 3 | 1 | 0 | 15.00 | 4 | 137 | 12 | 1.425 |
| A-7 | 8 | 1 | 0 | 8.25 | 15 | 456 | 13 | 0.750 |
| A-8 | 4 | 3 | 1.040 | 10.25 | 14.5 | 167 | 9 | 0.986 |
| A-9 | 7 | 6 | 1.748 | 17.29 | 26 | 350 | 11 | 0.902 |
| B-1 | 9 | 6 | 1.677 | 5.00 | 8 | 154 | 13 | 1.181 |
| B-2 | 2 | 2 | 0.693 | 2.50 | 175.5 | 13 | 3 | 0.721 |
| B-3 | 6 | 3 | 1.011 | 5.17 | 152 | 101 | 13 | 1.362 |
| B-4 | 2 | 2 | 0.693 | 7.00 | 105 | 53 | 10 | 1.407 |
| B-5 | 4 | 1 | 0.000 | 11.00 | 17.5 | 39 | 10 | 0.883 |
| B-6 | 4 | 1 | 0.693 | 1.5 | 8 | 130 | 10 | 1.408 |
| B-7 | 18 | 7 | 1.874 | 5.61 | 10 | 346 | 17 | 1.301 |
| C-1 | 4 | 3 | 1.039 | 17.50 | 21 | 194 | 10 | 1.245 |
| C-2 | 14 | 7 | 1.907 | 9.42 | 22.5 | 618 | 10 | 1.545 |
| C-3 | 10 | 2 | 0.325 | 8.90 | 25 | 602 | 15 | 1.001 |
| C-4 | 3 | 2 | 0.637 | 11.00 | 11 | 244 | 10 | 1.656 |
| C-5 | 6 | 3 | 1.055 | 5.80 | 17 | 476 | 9 | 1.255 |
| C-6 | 5 | 4 | 1.330 | 8.83 | 13 | 400 | 14 | 1.584 |

The organisations and groups have been given codes to anonymise the source.
