# Supplementary material for: Interdisciplinary collaboration from diverse science teams can produce significant outcomes
Source: PLoS One. 2022 Nov 29;17(11):e0278043. doi: 10.1371/journal.pone.0278043 (PMC9707800; doi:10.1371/journal.pone.0278043)
Supplement: S3 Appendix — (DOCX) [file pone.0278043.s003.docx]

**S3 Appendix. Responses to questions about perceived effectiveness and satisfaction with the group by respondents.**

| Group | response rate (%) | perceived group effectiveness (mean ± se) | satisfaction with group function (mean ± se) |
| --- | --- | --- | --- |
| A-1 | 36.36 | 4.4 ± 0.22 | 4.2 ± 0.34 |
| A-2 | 33.33 | 4.4 ± 0.22 | 4.2 ± 0.34 |
| A-3 | 41.67 | 4.25 ± 0.42 | 4.25 ± 0.42 |
| A-4 | 35.71 | 4.8 ± 0.18 | 4.6 ± 0.22 |
| A-5 | 41.18 | 4.57 ± 0.28 | 4.57 ± 0.28 |
| A-6 | 30.00 | 3.4 ± 0.36 | 3.6 ± 0.22 |
| A-7 | 38.46 | 3 ± 0.56 | 3.8 ± 0.44 |
| A-8 | 30.77 | 4.5 ± 0.56 | 3 ± 0.61 |
| A-9 | 29.41 | 4 ± 0 | 3.5 ± 0.25 |
| B-1 | 35.29 | 4 ± 0.24 | 3.83 ± 0.15 |
| B-2 | 29.17 | 4.5 ± 0.25 | 4.13 ± 0.45 |
| B-3 | 27.78 | 4.2 ± 0.33 | 4.4 ± 0.22 |
| B-4 | 31.58 | 4.6 ± 0.22 | 4 ± 0.28 |
| B-5 | 20.00 | 3.67 ± 0.72 | 3.33 ± 0.54 |
| B-6 | 46.67 | 4.8 ± 0.18 | 4.5 ± 0.22 |
| B-7 | 56.25 | 4.5 ± 0.18 | 4.5 ± 0.25 |
| C-1 | 25.00 | 4.2 ± 0.18 | 4.2 ± 0.34 |
| C-2 | 68.18 | 4.63 ± 0.25 | 4.75 ± 0.23 |
| C-3 | 41.18 | 4.57 ± 0.28 | 4.43 ± 0.4 |
| C-4 | 53.57 | 4.14 ± 0.26 | 4.29 ± 0.26 |
| C-5 | 25.00 | 4.2 ± 0.34 | 4.4 ± 0.31 |
| C-6 | 25.00 | 4 ± 0.31 | 4.43 ± 0.26 |

The organisations and groups have been given codes to anonymise the source.
