# Supplementary material for: Interdisciplinary collaboration from diverse science teams can produce significant outcomes
Source: PLoS One. 2022 Nov 29;17(11):e0278043. doi: 10.1371/journal.pone.0278043 (PMC9707800; doi:10.1371/journal.pone.0278043)
Supplement: Attachment — Submitted filename: PLOS R1 reviews_221101.docx [file pone.0278043.s005.docx]

| **Reviewer comment** | **Response** |
| --- | --- |
| Reviewer #1: I greatly appreciate the authors' decisions in how they chose to address my comments, questions, and concerns. Overall, the paper is more effective in its goal and the main document does not have the same issues. I have no issues with this paper being accepted due to the enhanced literature review and additional analyses that were conducted at my recommendation. | Thank you! |
| Reviewer #2: This is a good paper, and one worth publishing. I appreciate the work the authors did to improve the paper in light of the previous round of comments. I think the results are especially interesting and helpful. I suspect I will cite this paper in the future. | Thank you! |
| R2.2) FWIW, I disagree with both parts of this reply to my previous comment on ll. 265ff:  "We think it unlikely that authors could draw on literature from different domains in a paper without offering any synthesis or integration, that is, to be multi-disciplinary in a single paper. The scenario suggested where members of the group publish in their home disciplines without any interdisciplinary integration would yield a high score for publication diversity but a low score for citation diversity, since the individual disciplinary papers would not need (or be able to) to cite outside the discipline, so that effect would be visible in our analyses."  I have been in more than one crossdisciplinary collaboration that generated papers which would appear interdisciplinary on the authors assumptions but were decidedly multidisciplinary in their construction: different authors were given responsibility for different sections, and they brought their references along with them. And also in my experience, if a group publishes papers in the home disciplines of their members while keeping those members as co-authors (e.g., if you have the same set of co-authors but different first authors), the publications will often share a multidisciplinary set of references in common. (Perhaps there are some disciplines whose journals discourage citation outside the discipline, but I have not encountered that.)  That said, the authors are correct that the bibliometric methods they use as proxies for interdisciplinary collaboration are standard in the literature. I think they are weak measures of interdisciplinary collaboration, and as such represent a substantial limitation of this paper, but I don’t think it is fair to hold that against the authors since they are cleaving to standard practice here. | We acknowledge in the paper that the measures are proxies that do not fully capture nuances of the collaboration process, but as the reviewer notes, they are measures that are commonly used in the field.  Nevertheless, given the emphasis on integrating different perspectives on data and research problems in the working group collaboration process (our research context), we believe that they do provide a reasonable measure for our groups. |
| One general comment: I find the numbering helpful in keeping straight how you are nesting the sections and subsections. Perhaps this is inconsistent with PLOS One style, though? At this point, I have difficulty keeping track of which are the main sections, which are the subsections, and which are the sub-subsections. | We agree that numbering is helpful, but PLOS papers do not seem to number the sections. |
| I do have a few small comments/suggestions, none of which are deal-breakers. By line number:  l. 85 Reference [5] is repeated in here as reference [29]. | We have corrected reference 5, which should have been:  Laursen BK, Motzer N, Anderson KJ. Pathway profiles: Learning from five main approaches to assessing interdisciplinarity. Research Evaluation. 2022; rvac036. doi:10.1093/reseval/rvac036  Number 29 remains O’Rourke et al. 2016. |
| ll. 112-3 This still seems too fast to me. You can have a diverse team with very little integration – e.g., a multidisciplinary team that has one person (say, the PI) who is especially committed to broad citation. “[T]he diversity of authors and the references they cite” give you very little information about whether any of the processes mentioned in the quote from [33] actually took place in the team. IMO, of course. (Not that you are in a position to do better than this, given that you weren’t part of these groups – see the comment above.) It would make me a bit happier to see the authors note the gap between the processes mentioned in the quote and the proxy variables mentioned in the last sentence. | We have added a sentence of disclaimer at the end of line 111, replacing the last sentence in the paragraph:  “The details of these interactions are subtle and difficult to capture in a comparable way across teams. Research has therefore usually adopted proxy measures. In this paper we assess interdisciplinary research output by the diversity of the authors and of the references they cite [34, 35].” |
| l. 185 Should be ‘have’, not ‘has’. | Corrected |
| ll. 205-8 I have difficulty parsing the clause that includes two instances of "accepted by" – is there something missing between "university" and the second "accepted"? | We have inserted “and complying sufficiently with” before “the Australian ethics standards” (we have an email to say this). |
| l. 372 Space missing between ‘members’ and ‘who’. | Added space. |
| ll. 479-81 On what is this claim based? | The claim is “overall the mix of respondents seems representative of scientists who participate in working groups”. This claim was based on the authors’ personal experience coordinating working groups.  We have added three relevant references to this claim [18, 38, 42], and a statement to support our personal observation: “and matchednthe second author’s personal experience coordinating working groups”. |
| l. 543 If you make reference to the section numbering, you should number your sections. | As noted above, PLoS papers seem not to number sections, so we replaced “is included in section 4.3” with “is included in the qualitative analysis”. |
| ll. 669-70 The two "but" clauses in this sentence makes it difficult to interpret. Rewrite? | Replaced “but” in line 670 with “and”. |
| ll. 682-3 Not sure how to read this last clause. Is it supposed to be another example of positive consideration of the value of diversity in general? Maybe set its context up a bit more? | For our ease of reference, the statement is “and the value of ‘having a diverse set of viewpoints to contribute to the work’”.  We have separated this comment from the prior quotation and provided context: “A member of another group, which comprised members from the non-research community, systems analysts, web developers, ecologists, and those concerned with management and social engagement, noted that “having a diverse set of viewpoints to contribute to the work” as a factor that contributed to the group’s success. |
| ll. 789-90 Would it be fair to say that your results only show muted short-term impact? | We have added the following disclaimer.  “We acknowledge that our citation data are relatively short-term (1 to 10 years after publication). Even though papers are usually cited most heavily shortly after publication, it’s possible that more interdisciplinary papers will go on to be more cited in the longer run, as suggested by the literature [51].”  An incidental edit: On line 789 the reference to Fig 2 should have been to Fig 3. |
| ll. 800-3 Perhaps this is not surprising, given that you only draw from groups that were productive and productivity is positively correlated with satisfaction (ll. 481-3). | We appreciate the comment, but we consider our comment to be reasonable as not all groups were productive, as can be seen from the Supplementary material (S2 Appendix), where output ranged from 2 publications to 24. 2 publications for such well-supported groups is not a good outcome.  We have modified the text in new lines 497–9 as further caveat:  “Groups that had little productivity at the time of the survey tended not to respond to the survey”. |
| ll. 868-70 Not sure what you mean by "staged output"? What findings suggest this? I am not sure I can locate what in your Results section supports this guideline. | This recommendation is a response to the findings from the test of H2(c), namely the (unsurprising) positive correlation between satisfaction and the number of publications. We note that the Working Group Workflow shown in Figure 2 suggests that the norm is for groups to spend time planning, organizing data, etc. with publications coming only later in the process. We instead suggest that teams strive to maintain satisfaction by planning to publish throughout their collaboration and have suggested a staged publication (journal and conference) output. We have added a contrast to the implication of Figure 2 to guideline 2 by rewording it as follows:  “encourage publication (in conferences or journals) throughout the group process, as group satisfaction improves with the number of publications (in contrast to the delayed publication implied in a typical Working Group Workflow)” |
| l. 874 You don't need the commas in (4) – they make it hard to parse. | Removed. |
